# 3D strength surfaces for ankle plantar- and dorsi-flexion in healthy adults: an isometric and isokinetic dynamometry study

**DOI:** 10.1186/s13047-016-0174-1

**Published:** 2016-11-10

**Authors:** Sara J. Hussain, Laura Frey-Law

**Affiliations:** 1 Department of Health and Human Physiology, The University of Iowa, E102 Field House, Iowa City, IA 52242 USA; 2 Department of Physical Therapy and Rehabilitation Science, The University of Iowa, 1-252 Medical Education Building, Iowa City, IA 52242 USA

**Keywords:** Isometric, Isokinetic, Dynamometry, Ankle, Torque, 3D strength surfaces, Plantar-flexion, Dorsiflexion, Static, Dynamic, Muscle strength, Peak torque

## Abstract

**Background:**

The ankle is an important component of the human kinetic chain, and deficits in ankle strength can negatively impact functional tasks such as balance and gait. While peak torque is influenced by joint angle and movement velocity, ankle strength is typically reported for a single angle or movement speed. To better identify deficits and track recovery of ankle strength after injury or surgical intervention, ankle strength across a range of movement velocities and joint angles in healthy adults is needed. Thus, the primary goals of this study were to generate a database of strength values and 3-dimensional strength surface models for plantarflexion (PF) and dorsiflexion (DF) ankle strength in healthy men and women. Secondary goals were to develop a means to estimate ankle strength percentiles as well as examine predictors of maximal ankle strength in healthy adults.

**Methods:**

Using an isokinetic dynamometer, we tested PF and DF peak torques at five joint angles (−10° [DF], 0° [neutral], 10° [PF], 20° [PF] and 30° [PF]) and six velocities (0°/s, 30°/s, 60°/s, 90°/s, 120°/s and 180°/s) in 53 healthy adults. These data were used to generate 3D plots, or “strength surfaces”, for males and females for each direction; surfaces were fit using a logistic equation. We also tested predictors of ankle strength, including height, weight, sex, and self-reported physical activity levels.

**Results:**

Torque-velocity and torque-angle relationships at the ankle interact, indicating that these relationships are interdependent and best modeled using 3D surfaces. Sex was the strongest predictor of ankle strength over height, weight, and self-reported physical activity levels. 79 to 97 % of the variance in mean peak torque was explained by joint angle and movement velocity using logistic equations, for men and women and PF and DF directions separately.

**Conclusions:**

The 3D strength data and surface models provide a more comprehensive dataset of ankle strength in healthy adults than previously reported. These models may allow researchers and clinicians to quantify ankle strength deficits and track recovery in patient populations, using angle- and velocity-specific ankle strength values and/or strength percentiles from healthy adults.

**Electronic supplementary material:**

The online version of this article (doi:10.1186/s13047-016-0174-1) contains supplementary material, which is available to authorized users.

## Background

The ankle is an integral part of the human kinetic chain, playing an important role in balance and gait [1, 2]. Deficits in multiple aspects of ankle muscle strength are common to ankle injuries [3], aging [4, 5], and disease processes such as diabetic neuropathy [6–8], and can negatively impact performance of many functional tasks. Ankle strength can be used clinically to identify increased risk for movement impairments, falls, and progression of neuropathy [9, 10] and as a marker of recovery during musculoskeletal rehabilitation after injury or surgical interventions [11, 12]. While the ankle involves multiple joints and is therefore capable of complex triplanar motion, ankle strength assessments have focused primarily on plantarflexion (PF) and/or dorsiflexion (DF) in healthy [5, 13–25] and patient [1, 2, 6, 7] populations. Indeed, dorsiflexion and plantarflexion strength in the sagittal plane have been associated with balance impairments, falls, and gait in patient populations [1, 2, 6, 9], but may also be more frequently reported due to the relative ease of measuring sagittal plane strength. However, in order to quantify ankle strength deficits seen in patients with ankle disorders of various etiologies and to examine ankle strength recovery following injury, a database detailing a range of normal static and dynamic ankle strength in healthy men and women, across movement velocities and angles, may be useful.

Maximum torque production about the ankle has been tested using isometric [21, 23, 24, 26] and isokinetic [16, 17, 25] methodologies, resulting in two-dimensional relationships: torque-angle or torque-velocity. However, torque varies with both joint angle and movement velocity [22, 27], thus comprehensive strength assessments should include both factors resulting in three-dimensional (3D) relationships. 3D models provide several advantages compared to simpler 2-dimensional (2D) torque-velocity and torque-angle relationships; namely, they enable the visualization and quantification of interactions between torque-velocity and torque-angle relationships, as has been reported previously in other joint systems [13, 27–29]. Thus, the need for a more comprehensive normative strength data at the ankle joint remains.

Increasingly, models of human capability have the potential for clinical utility. For example, forms of digital human modeling are incorporated into many gait analyses, estimating net joint torques during ambulation or other functional tasks. Advances in digital human modeling have increased the need for mathematical representations of muscle strength about a joint, allowing for estimation of task requirements as a function of maximum capability. Digital human models also rely on accurate strength models when used to predict strategies of human movement [27, 30]. Several models of knee, elbow, shoulder and wrist strength are available [27–29, 31, 32], but we are aware of only two ankle strength models in the literature, one of which involved only dynamic strength assessments [13, 22]. Further, these previous studies either did not allow for velocity-angle interactions [13], or resulted in relatively poor fits of ankle strength data at the group level [22]. We have previously demonstrated that nonlinear logistic equations are superior to polynomial equations when modeling 3D strength surfaces [33], but these equations have yet to be used for modeling ankle strength surfaces. Consequently, the primary aims of the current study were 1) to generate a database of ankle strength values for PF and DF in healthy men and women, and 2) to develop accurate 3-dimensional (3D) strength surface models for ankle PF and DF using nonlinear logistic models based on these data. Secondary aims of this study were to develop a means to estimate ankle strength percentiles in healthy adults and to examine predictors of maximal static and dynamic ankle strength in healthy adults, including height, weight, sex and self-reported physical activity levels as these may prove useful in future models of ankle strength.

## Methods

Fifty-three healthy participants between the ages of 18 and 45 years were recruited for this study (26 men, 27 women). Exclusion criteria included any significant history of musculoskeletal, cardiovascular, or neuromuscular disorders/abnormalities, pregnancy, current pain or use of any pain relieving medications (over-the-counter or prescription), as well as any history of major injury, trauma, or surgery to the lower extremities. Major psychiatric disorders, such as schizophrenia, were means for exclusion; however, participants taking anti-depressants or anti-anxiety medications were not excluded from the study. All participants provided their written, informed consent prior to participation in the study as approved by the University of Iowa’s Biomedical Institutional Review Board, and the study conformed to the standards set forth by the Declaration of Helsinki.

**Fig. 1:**
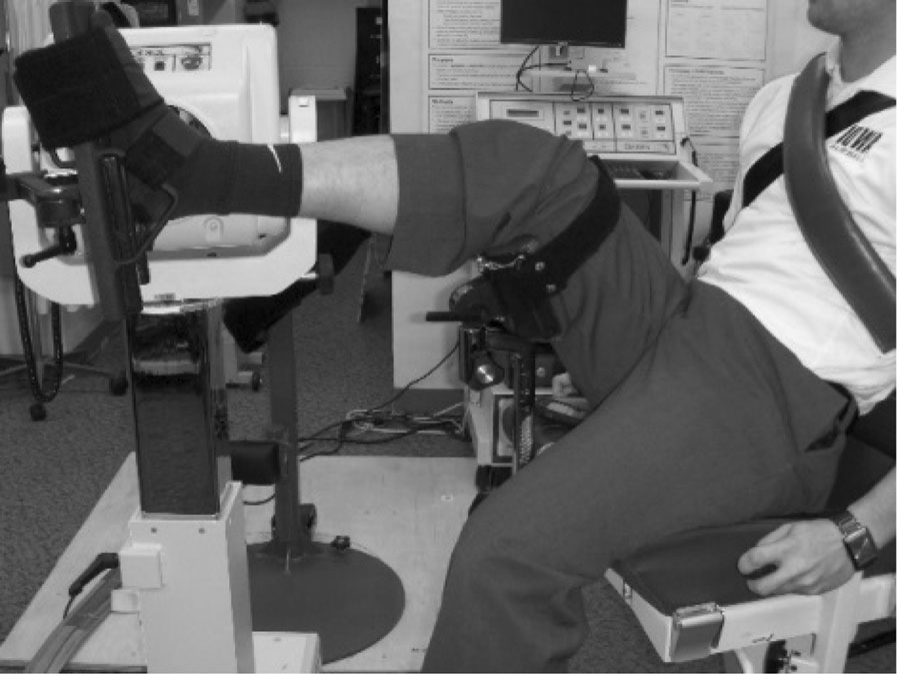
Experimental set-up for ankle strength testing using the Biodex isokinetic dynamometer with the knee flexed and ankle elevated

The study required a single visit, involving collection of demographic data (height, weight, and age), self-reported physical activity using the International Physical Activity Questionnaire (IPAQ long form) [34], and static and dynamic ankle strength measurements. After completing the surveys, participants performed 5 min of stationary cycling using both their arms and legs at a self-selected cadence (Schwinn Airdyne) as a generalized warm-up to minimize the risk of injury. Then, participants were seated in an isokinetic dynamometer (Biodex System 3.0, Biodex Medical Systems) with their right foot supported in the foot plate (see Fig. 1) following the standard Biodex recommendations for ankle strength assessment. The right ankle was tested for all participants, as between limb differences in ankle strength have previously been shown to be small. [15, 23] The right lower extremity was positioned with the hip and knees flexed to approximately 80° and 60°, respectively. The posterior thigh was fully supported by the standard Biodex padded attachment and secured with a Velcro strap. The lower leg was positioned parallel to the ground with the foot secured in the footplate, and the heel supported by a metal heel cup (attached to the foot plate) and secured using Velcro straps (see Fig. 1). While several straps and supports were used to minimize extraneous motion, due to soft-tissue compression during maximum efforts, not all extraneous motion can be eliminated. The axis of rotation of the dynamometer was aligned with the ankle center of rotation at approximately 2 cm distal to the lateral malleolus. Isometric testing was performed first, followed by the isokinetic testing. The order of the angles and/or velocities tested were block randomized to minimize possible order effects.

The first testing paradigm consisted of isometric plantarflexion (PF) and dorsiflexion (DF) at five joint angles, where negative angles were assigned to DF: −10° (DF), 0° (neutral), 10° (PF), 20° (PF), and 30° (PF). Neutral was defined as a 90° angle between the foot and the tibia. Each subject performed approximately 3 self-paced submaximal (< 50 % effort) warm-up contractions to familiarize themselves with the angle being tested. Following the warm-up repetitions and a brief rest interval (< 10 s), 3 five-second maximum voluntary isometric contractions (MVICs) were performed for the PF and DF directions at each angle. A rest interval of at least 30 s was provided between contractions at the same angle and 60 s between test angles. All participants were given directional cues (e.g. “push down” vs. “pull up”) with strong verbal encouragement throughout testing.

After completing all isometric assessments isokinetic PF and DF testing was performed at five different velocities: 30, 60, 90, 120, and 180 °/s. These velocities were tested in a block-randomized order to minimize order effects associated with fatigue. Before each new velocity, participants performed between 3 and 5 submaximal contractions, as needed, to familiarize themselves with the velocity being tested. During test contractions, participants were instructed to move their ankle through their full range of motion as fast as possible and to push and pull as hard as possible in each direction. Strong verbal encouragement was provided throughout testing. To ensure similar contraction times between different velocities, each subject performed between 3 and 10 isokinetic repetitions depending on velocity (i.e., more repetitions were performed at higher velocities). Participants rested for 3 min between test velocities.

Torque, position, and velocity analog outputs from the dynamometer were digitally sampled and recorded at 1000 Hz using a custom LabVIEW 8.0 program (National Instruments, Austin TX) and further analyzed using custom-written programs in Matlab (The MathWorks, Natick MA). Gravity correction was performed during post-processing for both isometric and isokinetic contractions by subtracting angle-specific passive torque values from the respective peak torques at each angle for isometric and isokinetic tests. These passive torque values were extracted from the torque recordings during the baseline quiet periods just prior to or following the isometric tests at each angle. Maximum angle-specific torque values were extracted from all trials (with passive torque subtracted from maximum values to account for the effects of gravity), and recorded as peak torque. To ensure isovelocity conditions, isokinetic peak torque extraction was limited to trials during which the subject achieved ± 15 % of the target velocity for each target angle (±2.5°). Thus, 30 angle-velocity specific peak torque values (5 positions and 6 velocities, including isometric) were extracted for both ankle PF and DF, generating a total of 60 data points per subject.

In cases of isolated missing data (i.e., if an isokinetic velocity was not within 15 % of the target velocity at a given angle), the missing data was individually modeled (imputed) for each subject using TableCurve 3D (Systat Software, Middlesex UK). This estimation prevented the non-random loss of the weakest and slowest individuals from biasing the resulting group mean values [27]. Cases of missing data occurred most frequently near the end of a subject’s range of motion and at faster velocities. However, if more than half of the male or female participants had missing data at a particular angle-velocity combination, that data point was not considered in the final surface analyses (men and women determined separately). Further, if any subject was missing more than two-thirds of the angle-velocity combinations, that subject’s data was also not included in the final surface analyses.

Descriptive statistics for peak torque (means and standard deviations) were calculated for male and female cohorts (SPSS 19.0, IBM, Armonk NY). Average coefficients of variation (CV) were computed for PF and DF torque values for men and women, separately. 3D mean peak torque surfaces (torque vs. angle vs. velocity) were plotted with separate surfaces generated for female PF, male PF, female DF and male DF. For strength data, we tested for significant effects of angle and velocity, as well as any interactions across angles (−10°, 0°, 10°, 20°) and velocities (0, 30, 60 and 120 °/s). These angles and velocities were chosen for statistical analyses as they represent the mid-range angles and velocities with the least number of missing data points. Thus, a 2 (sex) x 2 (direction) x 4 (velocity) x 4 (angle) mixed-model analysis of variance (ANOVA) was used to test for significant differences, with repeated measures on direction, velocity and angle. Huynh-Feldt correction was applied where appropriate to correct for non-sphericity.

Mathematical models of the 3D strength surfaces (male and female, PF and DF) were generated by fitting the mean torque values with a logistic equation using TableCurve3D (see Eq. 1). Logistic equations have been previously found to provide more physiologically feasible strength predictions than polynomials when extrapolated to joint ranges of motion beyond those angles tested [33]. Secondary analysis was performed to model plantarflexion strength surfaces without select isometric data points (see results) that may have been contaminated by hip extension moments. Thus, model fits of the full data set as well as a reduced data set were performed for plantarflexion surfaces only.

1 $$ \boldsymbol{P}\boldsymbol{T}\left(\boldsymbol{x},\boldsymbol{y}\right)=\boldsymbol{A}+\frac{\mathbf{4}\boldsymbol{B}{\boldsymbol{e}}^{\frac{-\left(\boldsymbol{x}-\boldsymbol{C}\right)}{\boldsymbol{D}}}}{{\left(\mathbf{1} + {\boldsymbol{e}}^{\frac{-\left(\boldsymbol{x}-\boldsymbol{C}\right)}{\boldsymbol{D}}}\right)}^2} + \frac{\mathbf{4}\boldsymbol{E}{\boldsymbol{e}}^{\frac{-\left(\boldsymbol{y}-\boldsymbol{F}\right)}{\boldsymbol{G}}}}{{\left(\mathbf{1} + {\boldsymbol{e}}^{\frac{-\left(\boldsymbol{y}-\boldsymbol{F}\right)}{\boldsymbol{G}}}\right)}^2} + \frac{\mathbf{16}\boldsymbol{H}{\boldsymbol{e}}^{\frac{-\left(\boldsymbol{x}-\boldsymbol{C}\right)}{\boldsymbol{D}}-\frac{\left(\boldsymbol{y}-\boldsymbol{F}\right)}{\boldsymbol{G}}}}{{\left(\mathbf{1} + {\boldsymbol{e}}^{\frac{-\left(\boldsymbol{x}-\boldsymbol{C}\right)}{\boldsymbol{D}}}\right)}^2\ast {\left(\mathbf{1} + {\boldsymbol{e}}^{\frac{-\left(\boldsymbol{y}-\boldsymbol{F}\right)}{\boldsymbol{G}}}\right)}^2} $$

Where: PT = peak torque (Nm),

x = joint angle (°),

y = angular velocity (°/s), and

A-H are fitted strength model parameters (see Table 2).

**Table 1:** Summary demographic characteristics of the full study population

Sex	Height (cm)	Weight (kg)	Age (yrs)	Activity (MET*min/week)
Males (*N* = 26)	179.8 (9.4)	83.4 (12.9)	27.1 (6.6)	7077 (5094 – 10754)
Females (*N* = 27)	166.1 (5.3)	62.9 (11.0)	29.5 (9.0)	5988 (2378 – 9377)
p-value*	**< 0.001**	**< 0.001**	0.27	0.16

*Significant differences between sexes are highlighted in bold; independent *t*-tests for height, weight, and age; non-parametric Mann–Whitney *U*-test for activity. Note: Mean (SD) reported for height, weight and age; median (25^th^ – 75^th^ interquartile range) reported for activity

**Table 2:** 3D strength surface model parameters for unadjusted and adjusted surfaces, with resulting R^2^ values, for women (unshaded) and men (shaded)

Strength Surface	Logistic Equation Parameter Values								R^2^
	A	B	C	D	E	F	G	H	
Female DF	−65.35	79.29	23.02	43.44	1056.06	−295.86	66.86	−762.36	0.95
Male DF	−54.81	78.02	17.85	29.12	372.89	−223.82	55.01	−16.93	0.97
Female PF	−9.72	17.85	4.14	16.95	59.16	−329.37	98.66	264.20	0.92
Male PF	−38.28	68.43	0.73	−23.68	−1042.06	−262.33	52.77	2628.56	0.89
Adjusted^a^ Female PF	−4.87	12.45	6.99	11.61	19.09	−65.52	78.43	27.85	0.98
Adjusted^a^ Male PF	−41.94	46.95	4.14	18.66	74.29	−584.05	185.18	291.76	0.95

^a^Plantarflexion (PF) surface models adjusted by removing two isometric peak torque values (0°, −10°) that may have been influenced by knee and hip extension moments. Note the increase in R^2^ values for the adjusted surfaces relative to the non-adjusted surfaces

To examine predictors of isometric and isokinetic torque in both PF and DF directions, we first calculated composite measures of isokinetic and isometric torque for both PF and DF. For isometric torque, we averaged torque values collected at 10, 20 and 30° PF for PF and DF directions separately. For isokinetic torque, we averaged torque values collected at 15 angle-velocity combinations (−10° DF, 0° PF, 10° PF, 20° PF and 30° PF at 30, 60 and 120°/s) for both PF and DF directions separately. Separate multiple regression analyses were then performed for isokinetic DF, isokinetic PF, isometric DF and isometric PF using the composite torque values. Predictors tested included height (cm), weight (kg), sex and physical activity levels (MET*min/week) as measured by the IPAQ. Secondarily, zero-order correlations between each predictor and each strength composite measure were performed as well as an ordered regression to estimate the contribution of each predictor to the total variance explained in each model (see supplemental materials). The IPAQ is a validated, self-report instrument used to assess physical activity over the past 7 days in multiple domains, including leisure, work, home, and transportation [34]. It asks individuals to estimate the number of days and number of minutes per day spent at different intensities (mild, moderate, or vigorous) for various activities within these four domains. We included an estimate of physical activity in addition to the more standard demographic variables as we hypothesized that each of these could have important influences on ankle strength. Significance was set as *p ≤ 0.05* for all comparisons.

Normative percentile scores for anthropometrics (such as height) are relatively commonplace and are useful for identifying deviations that lie outside of a normal range. However, the use of percentiles to describe normal peak strength variation in healthy and patient populations is currently uncommon. The availability of mean and SD strength data across a range of joint angles and movement velocities, and their respective mathematical models, allows for the unique ability to generate strength percentiles that may be useful for future sport, rehabilitation, and/or digital human modeling applications. Accordingly, standardized ankle strength *z*-scores can be estimated from modeled means and standard deviations or strength values for predefined percentiles can be calculated (see Eq. 2). We calculated 5^th^, 25^th^, 50^th^, 75^th^, and 95^th^ strength percentiles for men and women using the *z*-score values of −1.645, −0.675, 0, 0.675, and 1.645, respectively.

2 $$ P{T}_{\%}\left(x,y\right)=P{T}_{mean}\left(x,y\right) + \left(CV\ast P{T}_{mean}\left(x,y\right)\right)\ast z- score $$

Where: PT_%_ = Peak torque (Nm) for a specific percentile (e.g. 95^th^ percentile),

PT_mean_ = Model estimate or observed mean PT as function of angle and velocity,

CV = mean coefficient of variation for either PF or DF, and

*z*-score = standard *z*-score for a specific percentile (e.g., 1.645 for 95^th^ percentile).

## Results

A total of 53 healthy adults (26 men, 27 women) participated in the study. Ages ranged from 19 to 54 years and self-reported activity ranged from approximately 400 MET*min/week (sedentary) to 20,000 MET*min/week (highly vigorously active), but the majority of participants reported a high level of moderate activity in both men and women. See Table 1 for summary of demographic data.

**Fig. 2:**
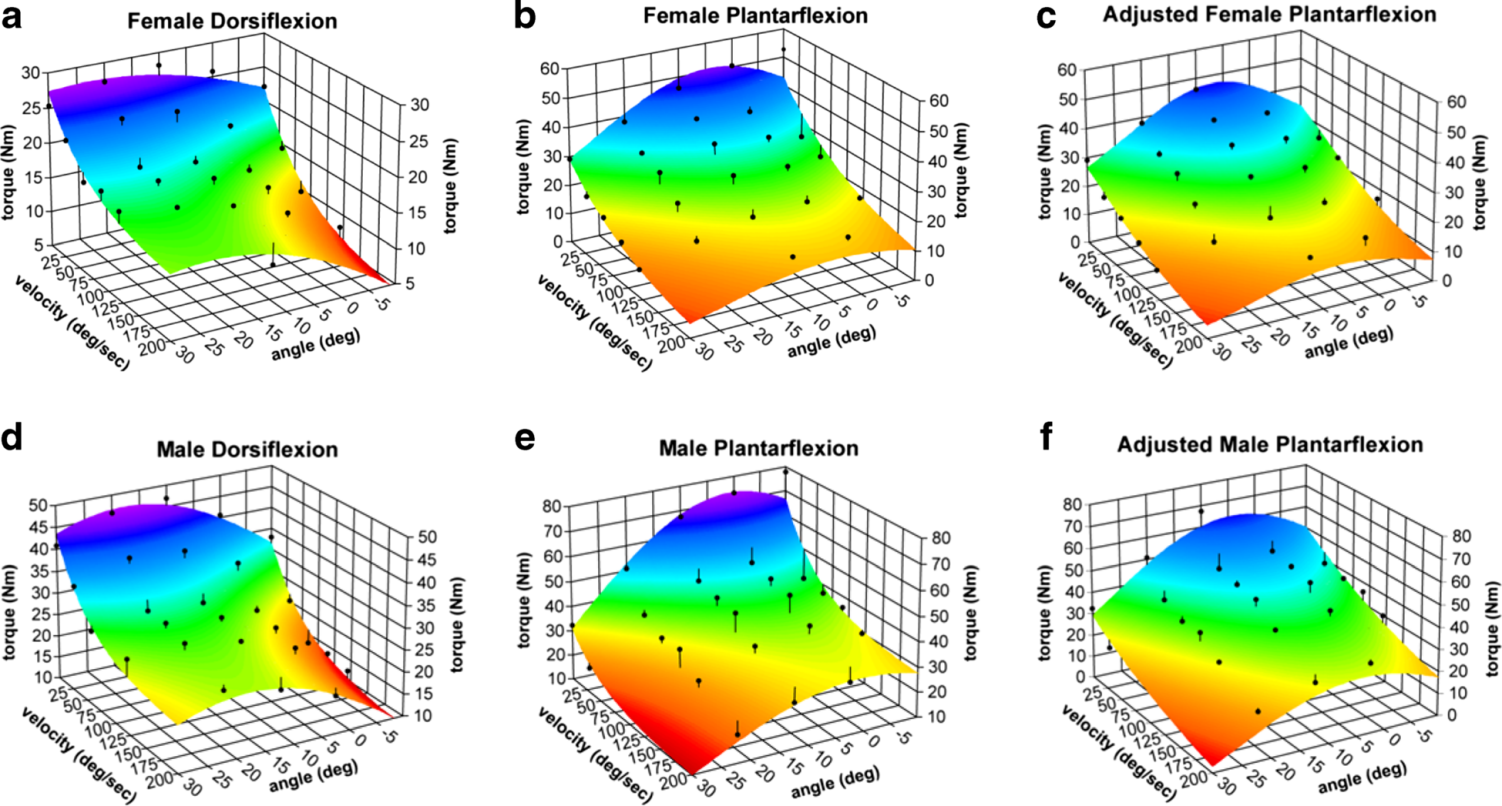
Mean peak torque values (Nm, black dots) for each angle-velocity combination with respective modeled 3D strength surfaces for (**a**) female DF, (**b**) female PF, (**c**) adjusted female PF (i.e., without 2 isometric angles, 0° and −10°, that may have included torque from knee and hip extension moments), (**d**) male DF, (**e**) male PF; and (**f**) adjusted male PF (i.e., without 2 isometric angles, 0° and −10°, that may have included torque from knee and hip extension moments)

Mean peak torque and the resulting 3D surface models are shown in Fig. 2a-d for males and females for both PF and DF (mean data also provided in supplemental Additional file 1: Tables S1 and Additional file 2: Table S2). All surface model parameter values and their resulting R^2^ values for the logistic equations are provided in Table 2. Two isometric data points appeared to have the highest likelihood of hip and knee extension torques contributing to the ankle torque measurement (data points corresponding to −10° and 0°). Accordingly, the plantarflexion strength surfaces fit without these two data points are shown in Fig. 2e and f and the model parameter fit vales included in Table 2. Overall, each mathematical model of the observed 3D strength surfaces explained between 92 and 97 % of variance observed in mean peak torque values.

Results from the four-way mixed-model ANOVA are presented in Table 3. PF torques were consistently higher than DF torques (*p < 0.001*). Peak torque varied with joint angle (*p < 0.001*) but the torque-angle relationship was different between PF and DF (significant angle x direction interaction, *p < 0.001*). That is, the peak torques occurred at opposite ends of the range of motion for PF and DF (see Fig. 2). Further, the torque-angle relationship varied across velocities (significant angle x velocity interaction, *p < 0.001*). Peak torque decreased with increasing velocity (*p < 0.001*). This decay in peak torque with increasing velocity differed between torque directions (significant direction x velocity interaction, *p < 0.001*), with a greater torque decline at faster speeds for DF compared to PF. As mentioned above, the significant angle x velocity interaction also indicates that the torque-velocity relationship varied across angles. Finally, ankle strength was also significantly higher in men than women (*p < 0.001*). When torque values were collapsed across all angles and velocities, males were on average 47 and 60 % stronger than females for PF and DF, respectively.

**Table 3:** Comparison of peak torque across angle, velocity, direction, and sex (N*  = 25 M, 23 F)

Factors	Df	*F* value	*p-value*
Sex	1,45	29.66	**< 0.001**
Direction (PF vs. DF)	1,45	77.98	**< 0.001**
Angle	3,135	24.65	**< 0.001**
Velocity	3,135	141.22	**< 0.001**
Direction x Sex	1,45	0.68	0.41
Angle x Sex	3,135	1.11	0.32
Velocity x Sex	3,135	4.67	**0.007**
Direction x Angle	3,135	70.59	**< 0.001**
Direction x Velocity	3,135	18.77	**< 0.001**
Angle x Velocity	9,405	16.27	**< 0.001**
Angle x Direction x Sex	3,135	12.93	**< 0.001**
Direction x Velocity x Sex	3,135	1.71	0.19
Angle x Velocity x Sex	9,405	0.61	0.64
Direction x Angle x Velocity x Sex	9,405	1.37	0.24

*Note total sample size was only 48, as 5 participants had missing data at one of the included data points, thus omitting them from the ANOVA analyses

Significant *p*-values indicated with bold typeface

**Table 4:** Linear regression models for predicting composite^a^ isometric and isokinetic peak torques

Model	Predictor	Coefficients		*p*-value
		Beta	Std. Beta	
Isometric PF				.077
(R^2^ = 0.174)	Height (cm)	-.146	-.089	.705
(*N* = 48)	Weight (kg)	.263	.228	.313
	Sex (M = 0,F = 1)	−8.886	-.271	.172
	Activity (MET^a^min/week)	.000	.086	.566
Isometric DF				**< .001**
(R^2^ = 0.683)	Height (cm)	.119	.110	.451
(*N* = 52)	Weight (kg)	.159	.226	.107
	Sex (M = 0,F = 1)	−12.481	-.562	**.000**
	Activity (MET^a^min/week)	.000	.044	.616
Isokinetic PF				**.004**
(R^2^ = 0.297)	Height (cm)	.470	.337	.124
(*N* = 48)	Weight (kg)	-.116	-.119	.569
	Sex (M = 0, F = 1)	−9.263	-.333	.072
	Activity (MET^a^min/week)	-.001	-.202	.147
Isokinetic DF				**< .001**
(R^2^ = 0.704)	Height (cm)	.121	.193	.172
(*N* = 52)	Weight (kg)	.142	.350	**.012**
	Sex (M = 0,F = 1)	−5.031	-.395	**.001**
	Activity (MET^a^min/week)	.000	-.006	.941

^a^Composite isometric torques were calculated as the means from 10, 20 and 30° PF for each direction; Composite isokinetic torques were calculated as the means from 15 angle-velocity combinations (−10° DF, 0° PF, 10° PF, 20° PF and 30° PF at 30, 60 and 120°/s) for each direction

Note 4 subjects had missing data for PF torque, but not DF torque, thus sample sizes for these composite strength score analyses are not equal

Significant *p*-values are indicated with bold

Regression analyses revealed that the combination of height, weight, sex and self-reported physical activity levels explained a greater proportion of variance in DF than PF strength (Table 4). These predictors collectively explained 68.3 % of the overall variance in isometric DF torque (*p < 0.001*) and 70.4 % of the overall variance in isokinetic DF torque (*p < 0.001*). For both isometric and isokinetic DF torque, sex was the main predictor even after controlling for height, weight, and activity levels (*p < 0.002* for both). Weight significantly predicted isokinetic DF torque values only (*p = 0.012*), after sex was already accounted for. However, because height and weight also differed between men and women, these collinear variables each can explain a large proportion of the variance in strength in isolation, but offer little additional information collectively (See Additional file 3: Tables S3 and Additional file 4: Table S4 for additional correlation and ordered regression results). For PF, the combination of these variables did not significantly predict isometric torque values (R^2^ = 0.174; *p = 0.077*) although it predicted 29.7 % of isokinetic torque values (*p = 0.004*). In isolation, sex and weight were significantly related to isometric PF torque (R = −.38, *p = 0.009*, and R = .35, *p = 0.015*, respectively; see Additional file 3: Table S3), but when all four predictors were included in the model, sex was the only variable that approached significance (*p = 0.072*). In sum, sex was the strongest predictor of PF torque in general even after adjusting for height, weight and self-reported physical activity levels.

Average CVs for PF (0.51, 0.49) were consistently larger than for DF (0.29, 0.26) in both men and women, respectively. Examples of strength percentiles for select angles and velocities are provided in Additional file 5: Table S5 using the mean and CV information as indicated in Eq. 2.

## Discussion

This study provides a comprehensive database of ankle PF and DF peak torque as a function of joint angle and movement velocity in healthy men and women, and resulting 3D strength surface models. These data and the associated models may be useful for estimating average peak torques for a range of joint angle and movement velocity conditions. This information may be relevant for sport, rehabilitation, and digital human modeling applications as it provides the most comprehensive assessment of ankle strength in healthy men and women to date. While estimates of normative ankle isometric or isokinetic peak strength have been previously reported [14, 17, 23, 35, 36], these estimates often do not account for the nonlinearities that occur when assessing peak torque across both multiple angles and velocities. As demonstrated both here and previously [33], peak torque 3D surfaces reveal nonlinear relationships with angle and velocity that can largely be represented using logistic equations.

Although this study provides a more comprehensive evaluation of peak torque about the ankle, the torque-angle relationships observed here are similar to those observed previously, supporting the validity of these strength surfaces. Two previous studies also found isometric DF peak torque values around 50 Nm that occurred at angles of approximately 15° PF [13, 26]. Both studies had sample sizes of 7 per cohort (young males only, [26]; young and old men and women, [13]). While it is not unexpected to find isometric PF torque to be consistently higher than DF torque [4, 13], the large difference observed in our study, particularly at smaller ankle angles, may have been influenced by hip extension moments. The standard Biodex set-up for ankle testing includes a thigh support to minimize extraneous movement. However, when the ankle is in neutral or a dorsiflexed posture, it is difficult to ensure full ankle isolation with no hip or knee extension torque production during a maximum voluntary contraction of the plantarflexor muscles. That is, when participants tried to “push the pedal” maximally, torque generated at the knee and hip may have contributed to the torque measured at the ankle. To address this possibility, we generated 3D surfaces and mathematical models without these potentially affected data points. In our laboratory, we have also observed that participants are better able to isolate a given joint during isokinetic compared to isometric contractions; thus, the use of hip and knee extension to help augment peak torques seemed to be less of an issue during isokinetic testing. Although we modeled the strength surfaces both with and without data points that were potentially influenced by hip extension moments, it is still possible that hip extension moments could have contributed to torques measured at other joint angles in more subtle ways.

Similarly, our 3D surface models of peak ankle torque are consistent with prior two-dimensional torque-velocity relationships. The decay in peak torque with increasing movement velocity has been consistently observed throughout the literature within nearly all joint systems [13, 22, 27–29, 31]. We observed a torque-velocity decay that was approximately 50 % from isometric to the fastest isokinetic speeds tested, which is consistent with prior reports [13, 25]. One of the most notable differences between our study and previous ankle strength reports is our ability to assess for a significant interaction between torque-velocity and torque-angle relationships. That is, the torque-velocity relationship varies across joint angle, and the torque-angle relationship varies with velocity. These findings strongly suggest that torque-angle and torque-velocity relationships are interdependent and are best represented using 3D surfaces, consistent with findings at the ankle using only isokinetic testing [22] and other joint systems [27–29, 31].

The unique shapes of the 3D ankle PF versus DF strength surfaces are likely primarily due to the underlying biomechanical properties of muscle length-tension and muscle moment arm variations with joint angle as well as the torque-velocity relationships due to contractile properties of muscle [37]. This is supported by the qualitatively similar surfaces between sexes, with few (only 2 of the 7) interactions including sex reaching significance: velocity x sex and angle x direction x sex. This is consistent with our prior findings at the knee and elbow [27], where only minor differences in the shape of the 3D surfaces were present between men and women, but large differences were apparent between torque directions. Overall, men exhibited greater ankle strength than women for both PF and DF. Yet, only a few other studies have included and/or reported separate ankle torque values for men and women: Anderson and colleagues (2007) tested ankle strength in men and women but only reported model parameters and not mean strength data [13], whereas Fugl-Meyer evaluated trained and untrained men and women, finding that men achieved on average from 35–55 % larger isokinetic ankle strength values than women [14, 15]. Khalaf et al. [22] also found a significant sex difference in ankle strength, but the magnitude of that difference was not reported [19]. Thus, the general trend of men exhibiting stronger ankle torque values is consistent across studies.

A secondary goal of the current work was to evaluate demographic predictors of ankle strength, although only sex reached significance, particularly for dorsiflexion strength. Anthropometric variables such as height and weight did not explain additional between-subject variance in ankle strength, except for isokinetic dorsiflexion, where weight was a significant, albeit small, contributor. This is in agreement with some previous findings that body anthropometry explains only a small proportion of muscle strength [38], particularly at the ankle [19]; but in opposition to other findings that a composite body area measure (from height and weight) [15] or age and weight independently [35] were associated with ankle plantar flexion strength. Lastly, self-reported physical activity did not further explain ankle strength variance in our cohort, contrary to our initial expectations. In this study, self-reported physical activity levels were addressed through the IPAQ, which asks the individual to estimate the amount of time they spent during the last 7 days doing various physical activities (such as walking to and from work, gardening, performing household chores) at different intensities (i.e., moderate, vigorous). Accordingly, these results suggest that individuals participating in greater overall physical activity display no greater ankle strength on average than those who report less physical activity. However, the IPAQ is a self-report measure of the amount of physical activity an individual performs across multiple domains in their daily lives, and does not focus strictly on either lower-extremity activities or specific exercise habits. Although it is well-documented that strength training can increase torque production, the current results may be consistent with a prior study showing regular endurance activity does not result in similar gains in peak ankle strength [39].

The strength models presented here could be used to generate strength percentiles for healthy men and women, in order to more precisely estimate and quantify ankle strength declines in patient or aging populations. One important implication for using these data to generate percentiles is that we observed a larger coefficient of variation (CV) for PF than DF. This indicates that the range of normal strength (i.e., from 5^th^ to 95^th^ percentiles) is wider for PF than DF in healthy men and women, and may be an important consideration when assessing whether an individual’s strength levels are within a normal range. This is consistent with a previous finding that PF strength differs more between athletes and untrained individuals than DF strength [14]. While this is the first study to provide comprehensive estimates of ankle strength means and standard deviations across a range of angles and velocities, this data is not able to indicate which percentile (i.e., level of strength deficit) best indicates a pathological or dysfunctional status.

Several limitations to the current study are worth noting. First, we only tested ankle strength at one knee angle in the current study. Given that the gastrocnemii cross both the ankle and knee joints and contribute significantly to PF torque production [20], changes in the degree of knee flexion can influence maximum PF torque measured at the ankle. Second, as in all studies of maximal voluntary torque production, we cannot be sure that participants truly exerted maximal efforts during testing, although we provided strong verbal encouragement in order to minimize this possibility. Third, although we assessed physical activity levels over the last 7 days through the IPAQ, we did not specifically assess participants’ physical activity in the 24 h preceding testing, so some variation in fatigue levels may have occurred across participants.

## Conclusion

This is the first study to report normative ankle PF and DF strength values along with mathematical models of these 3D ankle strength surfaces, incorporating both isometric and isokinetic strength assessments. This information is a first-step in developing a means for clinicians to better identify subtle deviations in strength from normal interactions between the torque-angle and torque-velocity properties, compared to single point estimates of ankle strength previously reported in the literature. The mathematical models presented here also allow the prediction of peak torques at ranges of motion and velocities beyond those tested in the current study, increasing the potential usefulness of this data. These findings are potentially relevant to sport, rehabilitation, and digital human modeling applications, providing a first step in the advancement of normative strength comparisons for healthy and patient populations.

## Additional files

Additional file 1: Table S1. Mean (SD) ankle plantarflexion peak torque values (Nm) for males (shaded) and females (unshaded). (DOC 44 kb)
Additional file 2: Table S2. Mean (SD) ankle dorsiflexion peak torque values (Nm) for males (shaded) and females (unshaded). (DOC 43 kb)
Additional file 3: Table S3 Pearson correlation coefficients (*p-value*) between composite* isometric and isokinetic peak torques and four predictor variables. (DOC 29 kb)
Additional file 4: Table S4. Ordered linear regression models for predicting composite* isometric and isokinetic peak torques, starting with sex, then adding weight, height, and finally activity level. (DOC 55 kb)
Additional file 5: Table S5. Examples of ankle strength values (Nm) by percentiles, calculated from dorsiflexion and plantarflexion peak torque values (using Eq. 2) for males (shaded) and females (unshaded). (DOC 38 kb)

## Abbreviations

3D: Three dimensional
CV: Coefficient of variation
DF: Dorsiflexion
MET: Metabolic equivalent (3.5 mL O_2_/kg/min)
PF: Plantarflexion
PT: Peak torque
