# Supplementary material for: 3D strength surfaces for ankle plantar- and dorsi-flexion in healthy adults: an isometric and isokinetic dynamometry study
Source: J Foot Ankle Res. 2016 Nov 10;9:43. doi: 10.1186/s13047-016-0174-1 (PMC5105238; doi:10.1186/s13047-016-0174-1)
Supplement: Additional file 1: Table S1. — Mean (SD) ankle plantarflexion peak torque values (Nm) for males (shaded) and females (unshaded). (DOC 44 kb) [file 13047_2016_174_MOESM1_ESM.doc]

Table S1. Mean (SD) ankle plantarflexion peak torque values (Nm) for males (shaded) and females (unshaded).

|  |  | **Ankle Angle (°)** | | | | | | | | | | | |
| --- | --- | --- | --- | --- | --- | --- | --- | --- | --- | --- | --- | --- | --- |
|  |  | -10 DF | | 0 | | 10 PF | | 20 PF | | | 30 PF | | |
| **Velocity (°/sec)** | 0 | 77.8* | (25.4) | 73.2* | (15.3) | 67.4 | (21.7) | 50.7 | (17.7) | 32.3 | | (14.7) |  |
|  |  | 52.7* | (18.5) | 50.4* | (15.9) | 46.2 | (18.1) | 38.1 | (17.9) | 29.2 | | (9.8) |  |
|  | 30 | 37.9 | (25.8) | 48.8 | (19.8) | 45.9 | (17.7) | 36.6 | (17.5) | 19.9 | | (12.7) |  |
|  |  | 25.4 | (15.8) | 38.1 | (18.1) | 39.3 | (14.7) | 31.3 | (12.2) | 20.3 | | (9.2) |  |
|  | 60 | 36.3 | (27.7) | 47.0 | (24.3) | 43.9 | (19.4) | 32.3 | (15.7) | † | |  |  |
|  |  | 22.2 | (13.6) | 33.1 | (15.3) | 34.6 | (13.0) | 28.8 | (10.1) | 17.7 | | (9.1) |  |
|  | 90 | 35.4 | (21.8) | 45.2 | (22.1) | 42.6 | (21.7) | 33.1 | (18.7) | † | |  |  |
|  |  | † |  | 27.0 | (13.2) | 28.0 | (11.9) | 22.6 | (9.8) | 13.9 | | (8.2) |  |
|  | 120 | 29.8 | (21.7) | 37.7 | (21.3) | 34.4 | (19.3) | 25.7 | (15.6) | † | |  |  |
|  |  | 16.2 | (9.7) | 19.2 | (11.8) | 18.1 | (10.8) | 14.4 | (9.5) | 9.4 | | (6.5) |  |
|  | 180 | † |  | 25.5 | (13.7) | 22.9 | (12.7) | 16.0 | (10.7) | † | |  |  |
|  |  | † |  | 16.2 | (9.9) | 14.1 | (9.6) | † |  | † | |  |  |

† Missing data in > 50% of participants. Note: negative angles represent dorsiflexed postures, 0° = neutral PF/DF; and positive angles represent plantarflexed postures.

* Plantarflexion angles most likely influenced by hip extension moments (removed for adjusted surfaces and adjusted model fits).
