# Supplementary material for: 3D strength surfaces for ankle plantar- and dorsi-flexion in healthy adults: an isometric and isokinetic dynamometry study
Source: J Foot Ankle Res. 2016 Nov 10;9:43. doi: 10.1186/s13047-016-0174-1 (PMC5105238; doi:10.1186/s13047-016-0174-1)
Supplement: Additional file 2: Table S2. — Mean (SD) ankle dorsiflexion peak torque values (Nm) for males (shaded) and females (unshaded). (DOC 43 kb) [file 13047_2016_174_MOESM2_ESM.doc]

Table S2. Mean (SD) ankle dorsiflexion peak torque values (Nm) for males (shaded) and females (unshaded).

|  |  | **Ankle Angle (°)** | | | | | | | | | | | |
| --- | --- | --- | --- | --- | --- | --- | --- | --- | --- | --- | --- | --- | --- |
|  |  | -10 DF | | 0 | | 10 PF | | 20 PF | | | 30 PF | | |
| **Velocity (°/sec)** | 0 | 32.9 | (12.9) | 40.5 | (11.8) | 46.9 | (7.1) | 45.8 | (8.1) | 40.9 | | (11.5) |  |
|  |  | 22.1 | (7.0) | 25.8 | (7.0) | 28.1 | (7.5) | 27.2 | (7.7) | 25.4 | | (5.4) |  |
|  | 30 | 20.2 | (10.0) | 31.7 | (8.3) | 37.2 | (8.9) | 38.1 | (8.5) | 34.2 | | (9.0) |  |
|  |  | 14.4 | (4.0) | 19.3 | (4.1) | 23.0 | (4.8) | 23.5 | (4.5) | 22.0 | | (4.7) |  |
|  | 60 | 12.8 | (9.1) | 23.5 | (7.0) | 27.9 | (6.7) | 28.7 | (6.1) | 26.9 | | (7.1) |  |
|  |  | 9.6 | (3.6) | 14.6 | (3.1) | 17.4 | (3.3) | 18.3 | (3.4) | 17.9 | | (3.2) |  |
|  | 90 | 13.2 | (7.5) | 22.2 | (5.1) | 27.3 | (4.4) | 28.9 | (4.5) | † | |  |  |
|  |  | † |  | 13.8 | (2.9) | 16.8 | (3.5) | 18.1 | (4.0) | 18.4 | | (4.2) |  |
|  | 120 | 12.2 | (6.5) | 20.4 | (4.9) | 24.8 | (4.8) | 27.2 | (4.8) | 26.5 | | (6.0) |  |
|  |  | 8.0 | (3.4) | 11.9 | (2.6) | 14.7 | (3.1) | 16.2 | (3.9) | 17.5 | | (4.9) |  |
|  | 180 | † |  | 15.6 | (4.9) | 19.9 | (4.7) | 22.9 | (5.4) | † | |  |  |
|  |  | † |  | † |  | 10.2 | (5.6) | † |  | † | |  |  |

† Missing data in > 50% of participants. Note: negative angles represent dorsiflexed postures, 0° = neutral PF/DF; and positive angles represent plantarflexed postures.
