# Supplementary material for: 3D strength surfaces for ankle plantar- and dorsi-flexion in healthy adults: an isometric and isokinetic dynamometry study
Source: J Foot Ankle Res. 2016 Nov 10;9:43. doi: 10.1186/s13047-016-0174-1 (PMC5105238; doi:10.1186/s13047-016-0174-1)
Supplement: Additional file 3: Table S3 — Pearson correlation coefficients (p-value) between composite* isometric and isokinetic peak torques and four predictor variables. (DOC 29 kb) [file 13047_2016_174_MOESM3_ESM.doc]

Table S3. Pearson correlation coefficients (*p-value*) between composite* isometric and isokinetic peak torques and four predictor variables.

| **Predictors** | Isometric PF | Isometric DF | Isokinetic PF | Isokinetic DF |
| --- | --- | --- | --- | --- |
| Sex (M=0, F=1) | **-.38 (0.009)** | **-.79 (<0.001)** | **-.43 (0.002)** | **-.76 (<0.001)** |
| Weight (kg) | **.35 (0.015)** | **.69 (<0.001)** | **.31 (0.034)** | **.76 (<0.001)** |
| Height (cm) | .26 (0.077) | **.67 (<0.001)** | **.47 (0.001)** | **.73 (<0.001)** |
| Activity (MET*min/wk) | .19 (0.206) | .20 (0.15) | -.16 (0.292) | .14 (0.330) |

* Composite isometric torques were calculated as the means from 10, 20 and 30° PF for each direction; Composite isokinetic torques were calculated as the means from 15 angle-velocity combinations (-10° DF, 0° PF, 10° PF, 20° PF and 30° PF at 30, 60 and 120°/sec) for each direction.

Note: significant correlations are highlighted in bold (*p ≤ 0.05*)
