# Supplementary material for: 3D strength surfaces for ankle plantar- and dorsi-flexion in healthy adults: an isometric and isokinetic dynamometry study
Source: J Foot Ankle Res. 2016 Nov 10;9:43. doi: 10.1186/s13047-016-0174-1 (PMC5105238; doi:10.1186/s13047-016-0174-1)
Supplement: Additional file 4: Table S4. — Ordered linear regression models for predicting composite* isometric and isokinetic peak torques, starting with sex, then adding weight, height, and finally activity level. (DOC 55 kb) [file 13047_2016_174_MOESM4_ESM.doc]

Table S4. Ordered linear regression models for predicting composite* isometric and isokinetic peak torques, starting with sex, then adding weight, height, and finally activity level.

| Dependent variable | Models | R^2^ change | dof | Model *p-value* |
| --- | --- | --- | --- | --- |
| Isometric PF |  |  |  |  |
| Reduced model 1 | Sex (M=0,F=1) | .141 | 1,46 | **.009** |
| Reduced model 2 | add Weight (kg) | .021 | 2,45 | **.019** |
| Reduced model 3 | add Height (cm) | .006 | 3,44 | .043 |
| Full model 4 | add Activity (MET*min/wk) | .008 | 4,43 | .077 |
|  |  | **(total R^2^ = 0.174)** |  | .077 |
| Isometric DF |  |  |  |  |
| Reduced model 1 | Sex (M=0,F=1) | .631 | 1,50 | **<.001** |
| Reduced model 2 | add Weight (kg) | .048 | 2,49 | **<.001** |
| Reduced model 3 | add Height (cm) | .003 | 3,48 | **<.001** |
| Full model 4 | add Activity (MET*min/wk) | .002 | 4,47 | **<.001** |
|  |  | **(total R^2^ = 0.684)** |  | **<.001** |
| Isokinetic PF |  |  |  |  |
| Reduced model 1 | Sex (M=0,F=1) | .188 | 1,46 | **.002** |
| Reduced model 2 | add Weight (kg) | .002 | 2,45 | **.009** |
| Reduced model 3 | add Height (cm) | .071 | 3,44 | **.004** |
| Full model 4 | add Activity (MET*min/wk) | .036 | 4,43 | **.004** |
|  |  | **(total R^2^ = 0.297)** |  | **.004** |
| Isokinetic DF |  |  |  |  |
| Reduced model 1 | Sex (M=0,F=1) | .569 | 1,50 | **<.001** |
| Reduced model 2 | add Weight (kg) | .120 | 2,49 | **<.001** |
| Reduced model 3 | add Height (cm) | .014 | 3,48 | **<.001** |
| Full model 4 | add Activity (MET*min/wk) | .000 | 4,47 | **<.001** |
|  |  | **(total R^2^ = 0.703)** |  | **<.001** |

* Composite isometric torques were calculated as the means from 10, 20 and 30° PF for each direction; Composite isokinetic torques were calculated as the means from 15 angle-velocity combinations (-10° DF, 0° PF, 10° PF, 20° PF and 30° PF at 30, 60 and 120°/sec) for each direction.

Note the R^2^ change value is dependent on the order the predictors are entered as height, weight, and sex are related variables. The first one entered inherently explains the largest proportion of the variance in the composite strength score.
