# Supplementary material for: 3D strength surfaces for ankle plantar- and dorsi-flexion in healthy adults: an isometric and isokinetic dynamometry study
Source: J Foot Ankle Res. 2016 Nov 10;9:43. doi: 10.1186/s13047-016-0174-1 (PMC5105238; doi:10.1186/s13047-016-0174-1)
Supplement: Additional file 5: Table S5. — Examples of ankle strength values (Nm) by percentiles, calculated from dorsiflexion and plantarflexion peak torque values (using Eq. 2) for males (shaded) and females (unshaded). (DOC 38 kb) [file 13047_2016_174_MOESM5_ESM.doc]

Table S5. Examples of ankle strength values (Nm) by percentiles, calculated from dorsiflexion and plantarflexion peak torque values (using Eq 2) for males (shaded) and females (unshaded).

|  |  | **Percentile (%)** | | | | |
| --- | --- | --- | --- | --- | --- | --- |
|  |  | **5^th^** | **25^th^** | **50^th^** | **75^th^** | **95^th^** |
| **Angle (°) and Velocity (°/sec) condition** | DF  10° - 0°/s | 24.5 | 37.7 | 46.9 | 56.1 | 69.3 |
|  |  | 16.1 | 23.2 | 28.1 | 33.0 | 40.1 |
|  | PF  10° - 0°/s | 10.9 | 44.2 | 67.4 | 90.6 | 123.9 |
|  |  | 9.0 | 30.9 | 46.2 | 61.5 | 83.4 |
|  | DF  10° - 60°/s | 14.6 | 22.4 | 27.9 | 33.4 | 41.2 |
|  |  | 10.0 | 14.3 | 17.4 | 20.5 | 24.8 |
|  | PF  10° - 60°/s | 7.1 | 28.8 | 43.9 | 59.0 | 80.7 |
|  |  | 6.7 | 23.2 | 34.6 | 46.0 | 62.5 |
|  | DF  10° - 120°/s | 13.0 | 19.9 | 24.8 | 29.7 | 36.6 |
|  |  | 8.4 | 12.1 | 14.7 | 17.3 | 21.0 |
|  | PF  10° - 120°/s | 5.5 | 22.6 | 34.4 | 46.2 | 63.3 |
|  |  | 3.5 | 12.1 | 18.1 | 24.1 | 32.7 |

Note: positive angles represent plantarflexed postures, 10° PF angle data shown for three velocities: 0, 60, and 120°/s.
